# Evaluation of Active Intraoperative Nerve Monitoring in Severe Developmental Dysplasia of the Hip Patients Undergoing Total Hip Arthroplasty

**DOI:** 10.1016/j.artd.2024.101612

**Published:** 2025-01-20

**Authors:** Alireza Manafi Rasi, Sina Afzal, Mojtaba Baroutkoub, Hasan Shakiba, Pooya Kalani, Mehdi Tavassoli, Reza Zarei

**Affiliations:** a Department of Orthopedic Surgery, School of Medicine, Shahid Beheshti University of Medical Sciences, Tehran, Iran; b Pain Research Center, Neuroscience Institute, Tehran University of Medical Sciences, Tehran, Iran; c Department of Orthopedic Surgery, School of Medicine, Babol University of Medical Sciences, Babol, Iran

**Keywords:** Congenital hip dysplasia, Total hip arthroplasty, Epidural anesthesia, Sciatic nerve injury, Nerve monitoring

## Abstract

**Background:**

Total hip arthroplasty (THA) stands as the standard treatment in neglected developmental dysplasia of the hip (DDH), and its application in severe cases may be linked to debilitating outcomes, including nerve damage. Here, we aimed to report the results of intraoperative nerve monitoring (IONM) via an active method.

**Methods:**

In this retrospective cohort study, we recruited patients with Crowe types III and IV DDH, who underwent THA. The study comprised 2 cohorts: one without nerve monitoring and the other with active IONM under epidural anesthesia. The primary study outcomes included the incidence of neural complications, the extent of achieved leg lengthening, and the necessity for femoral osteotomy.

**Results:**

A total of 183 patients were included in this study as the cases underwent THA under epidural anesthesia and IONM, along with 156 historical cohorts of controls. In the group with IONM, no clinically postoperative nerve injury was detected, while in the control group, 6 (3.8%) patients experienced neural complications (*P* = .08). The mean achieved limb lengthening was significantly greater in the monitoring group as 4.2 cm (range = 2.4-5.6) than in the control group as 3.56 cm (range = 2.2-5.6) (*P* = .04). The rate of femoral osteotomy was significantly lower in the monitoring group (13.6%, 25/183) compared to the control group (27.5%, 43/156) (*P* < .005).

**Conclusions:**

The utilization of active IONM in patients under epidural anesthesia during THA for severe DDH proves to be an effective approach. This method allows for real-time assessment of nerve function throughout the surgical procedure, demonstrating its potential to minimize postoperative complications.

## Introduction

Developmental dysplasia of the hip (DDH) stands as a prevalent congenital disorder, imposing a significant burden of disability from childhood into adulthood [1]. DDH comprises the reason for about 10% of total hip arthroplasty (THA) and this number is as high as about 30% among individuals aged less than 60 years [2]. The clinical spectrum of DDH spans from minor acetabular dysplasia to severe, irreducible hip dislocation with accompanying femoral head displacement, compromising limb length and function [3]. Various severity classifications exist, with the Crowe classification, rooted in anatomic landmarks, notably the pelvis height, medial femoral head-neck junction, and pelvic teardrop, emerging as the most recognized and used [4,5]. The Crowe classification is based on some anatomic landmarks including the pelvis height, the medial femoral head-neck junction, and pelvic teardrop, which classifies the disease in 4 groups by percentages of hip dislocation (< 50%, 50%-75%, 75%-100%, and > 100%) and proximal displacement of femoral head of pelvic height (< 10%, 10%-15%, 15%-20%, and > 20%), among which the Crowe types Ⅲ and Ⅳ are the most severe forms [5].

Treatment of DDH depends on the patient’s age and the severity of the disease including nonsurgical methods in earlier ages and surgery in individuals generally aged 1.5-2 years which comprises open hip reduction with femoral osteotomy and pelvic osteotomy if needed [6]. THA is the gold standard and preferred surgical method in cases of end-stage DDH with osteoarthritis [7].

However, THA in DDH patients is not without challenges, with nerve injuries during limb length restoration, particularly to the sciatic nerve. The incidence of sciatic nerve injury is significantly higher in patients with DDH (5.8%) compared to primary THA patients (less than 1%) [8]. Such injuries manifest in varying degrees of neural damage, including reversible or irreversible nerve palsy [9,10]. Surgical maneuvers, such as anterior or medial limb movements, pose a risk to the sciatic nerve, with potential injuries surpassing those attributed solely to nerve traction [10,11]. Intraoperative nerve monitoring (IONM) is a beneficial method to monitor the nerve function during THA surgery and prevent further nerve damage by real-time assessment of the nerve function [12]. While various methods of nerve monitoring, such as electromyography and motor/sensory evoked potentials, have reported success in THA surgery for DDH and other hip pathologies, these studies predominantly use inactive nerve evokes in patients under general or spinal anesthesia [[12], [13], [14], [15], [16]]. This study seeks to explore the feasibility and outcomes of active nerve monitoring via toe and ankle movements in awake patients undergoing THA for severe DDH under epidural anesthesia, addressing a crucial gap in the existing literature.

## Material and methods

### Study design and population

We conducted a retrospective cohort study to examine the outcomes of active IONM under epidural anesthesia in patients with severe unilateral DDH undergoing THA. The study encompassed 2 orthopaedic surgery centers: Imam Hossein Hospital affiliated with Shahid Beheshti University of Medical Sciences and Atieh Private Hospital in Tehran, Iran.

The study included patients diagnosed with severe unilateral DDH (Crowe types III and IV) who underwent THA surgery. Two cohorts were established: a historical control cohort from September 2015 to October 2018, comprising surgeries without nerve monitoring, and a case cohort from October 2018 to October 2020, involving surgeries with active toe and ankle movement monitoring under epidural anesthesia.

Patients aged less than 60 years with DDH Crowe types III and IV were eligible for inclusion, provided complete data on THA surgery and outcomes were available in their admission records. Exclusions encompassed patients with incomplete surgery and outcome data, as well as those with epilepsy, cardiac problems, cerebral vascular hemorrhage, cerebral palsy, and poliomyelitis. Additionally, individuals who exhibited abnormal findings on preoperative nerve examination were excluded.

### Epidural anesthesia technique

After sedation with intravenous fentanyl (1 μg/kg) and midazolam (1 mg), skin cleansing with chlorhexidine, and excess removal, epidural anesthesia was performed with the patient in sitting position through the interlaminar parasagittal interspace L4-L5 in the side of surgery after skin infiltration with lidocaine 1%, using a 17G Tuohy needle. A single dose of bupivacaine 0.4% with 10 micrograms of sufentanil in total volume of 20 mL was injected after localization of the epidural space using the loss of resistance technique. Epidural catheters were threaded 5 cm cephalad.

After application of the epidural anesthesia, the patient was placed in a supine position, and an assessment of sensory block was performed after 20 minutes. The level of sensory block, defined as the lack of pinprick sensation, was assessed throughout the skin of the operating leg [17].

### Surgical technique

The primary objective across all surgeries was to achieve limb length equality to the greatest extent possible while reconstructing the acetabulum in its native position. All surgical procedures were exclusively conducted by a single surgeon (A. M. R.) employing the anterolateral approach in the lateral position.

Thorough neurological examinations were conducted for each patient by an orthopaedics chief resident both preoperatively and postoperatively. Nerve function assessments were undertaken 1 day before surgery, at multiple intervals during the operation, immediately postanesthesia recovery in the operating room, daily throughout hospitalization, and during routine follow-up visits.

**Figure 1:**
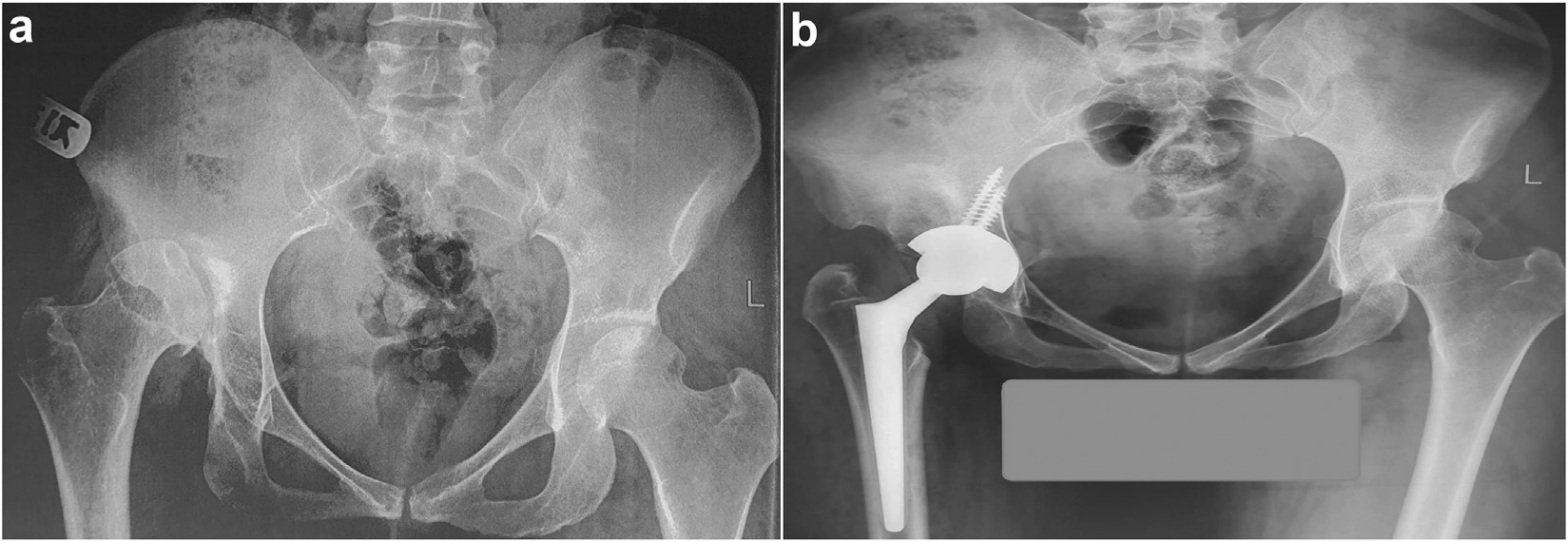
(a) Preoperative and (b) postoperative imaging of a patient with developmental dysplasia of the hip (DDH). In this case, trochanteric osteotomy was unnecessary due to the absence of neurological issues during intraoperative active neuromonitoring. Please note the equal length of both lower limbs in the postoperative X-ray.

In the monitoring group, there was no predetermined plan to conduct a subtrochanteric osteotomy (STO) (Fig. 1). Instead, the decision to proceed with STO was made in the event of challenges with hip reduction in maximum traction or the occurrence of nerve-related issues post hip reduction. Attaining the intended lengthening in these cases involved meticulous acetabular reconstruction in the anatomical position without the necessity for osteotomy.

### Intraoperative nerve monitoring

**Figure 2:**
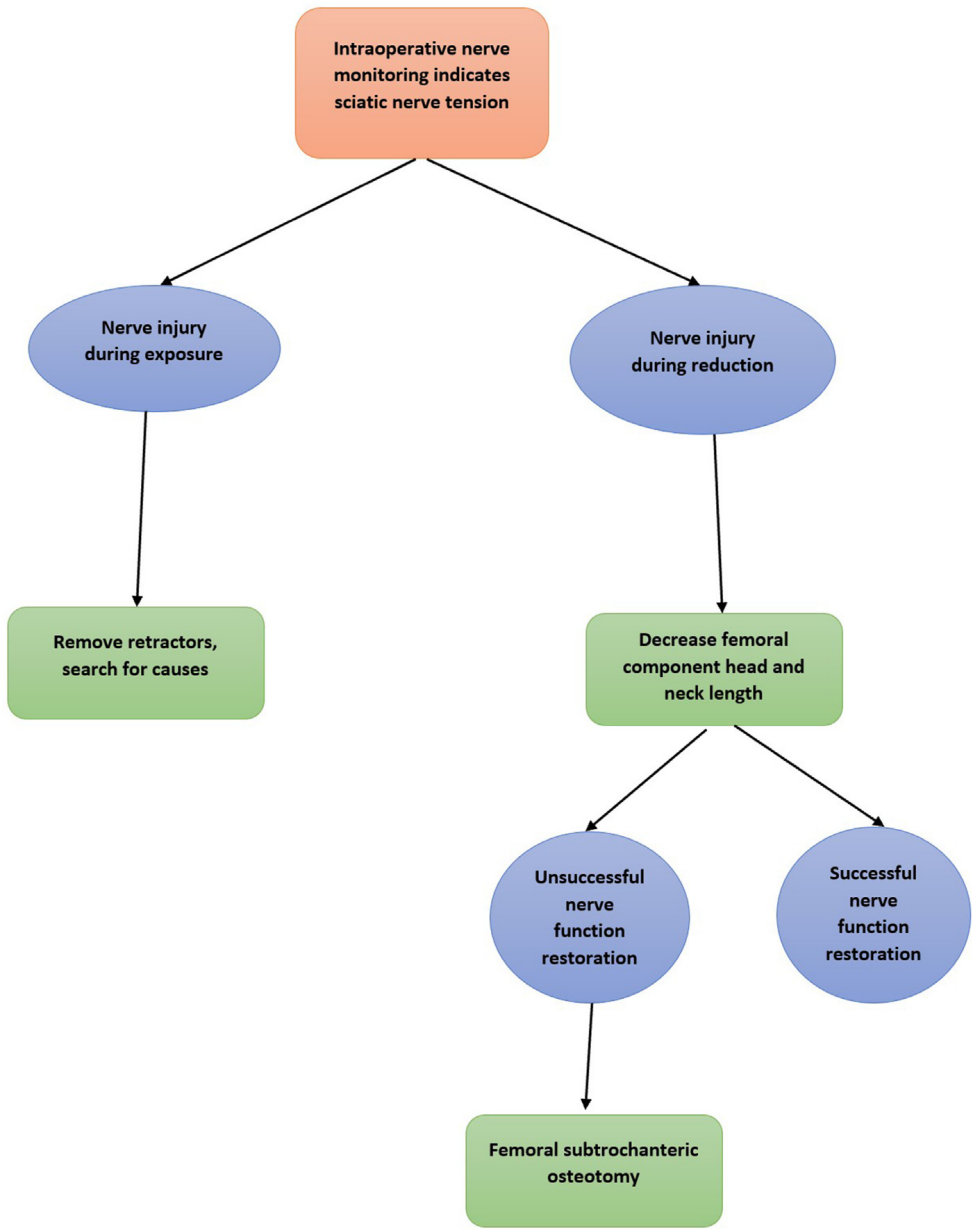
Algorithm for sciatic nerve injury management during total hip arthroplasty with active intraoperative nerve monitoring in patients with developmental dysplasia of the hip.

**Figure 3:**
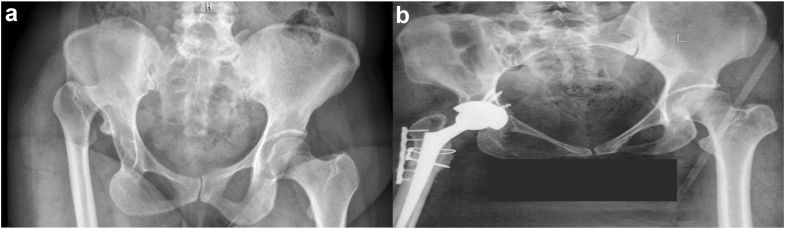
(a) Preoperative and (b) postoperative X-ray of a patient with Crowe IV developmental dysplasia of the hip (DDH). In this patient, due to neurological issues during the femoral trial reduction, it required additional bone resection from the femur before prostheses insertion.

IONM was performed multiple times throughout the whole surgical course by asking the patient to actively perform flexion and extension of ankle joint and toes. Any change in active movement or disability to actively perform flexion and extension in toes indicated that the sciatic nerve was under tension and at risk of injury. The surgeon then suspended the operation and reassessed the cause of the abnormal finding **(**Video. 1**)**. If the nerve injury occurred in the process of exposure, we first removed all retractors and searched for probable causes. If this situation occurred during the process of reduction, we decreased the length of the prosthetic femoral head and neck length. If this made no difference, the next step was a femoral STO (Fig. 2). In need, we performed transverse STO at 2 cm beneath the lesser trochanter. The actual length of the removed bone was measured using a ruler. If it still remained impossible to reduce the hip without a nerve injury, additional bone was gradually resected at the osteotomy site to reach satisfactory hip reduction and normal sciatic motor function. Again, following prophylactic wiring of the proximal and distal segments, sciatic motor function was checked, and the femoral prosthesis was implanted only when we had a normal sciatic motor function (Fig. 3). In the control group, our reference to perform STO or not was based on the soft-tissue tension. In presence of high-tension soft tissue, if reducing the hip with the reduced femoral head and neck length was not possible, STO shall be performed.

### Patient follow-up

Patients were followed up at regular intervals (3 weeks, 2 months, 4 months, and 12 months postoperatively), and clinical information was gathered at each visit.

### Study variables

Demographic variables of age and sex and anthropometric variables of weight and height were included in this study. The main outcomes of interest following surgery assessed in this study were the presence of sciatic nerve injury, the extent of limb lengthening during surgery (mm), and use of STO.

### Statistical analysis

Quantitative variables were summarized by mean with standard deviation, and categorical variables were summarized in frequency and percentages. Chi-squared test, Fisher’s exact test, and independent samples *t*-test were used to evaluate different categorical and quantitative variables between groups. *P* value < .05 was the statistical significance level in this study. IBM SPSS Statistics version 21 was used to analyze the data.

### Ethical considerations

This study received the ethical approval from the ethical committee at Shahid Beheshti University of Medical Sciences (code: IR.SBMU.RETECH.REC.1402.356). All patients provided written informed consent for conducted intervention and possible publication of the findings prior to surgery. Only study investigators had access to patients’ data and they were committed to save the privacy of patients in data collection, analysis, and reporting. The authors received no specific funding for this work.

## Results

### General findings

A total of 183 patients were included in this study as the cases underwent THA under epidural anesthesia and IONM, along with 156 historical cohorts of controls, based on the inclusion and exclusion criteria. Demographic and anthropometric factors of sex, age, height, weight, and body mass index showed no significant differences between study groups. STO was used 30 times. Augmentation of the roof by bone graft was needed in 8 hips using autologous bone.

### Intraoperative nerve monitoring findings

In the group with active nerve monitoring during surgery, no clinically detectible postoperative nerve injury was detected. In the control group, 6 (3.8%) patients experienced postoperative neural complications, among which 3 of the sciatic lesions affected only the peroneal division and 3 affected both the peroneal and tibial divisions. The tibial division was never affected alone. Two of the nerve injuries were complete, 3 showed only a partial motor deficit, and 1 showed a partial motor and sensory deficit.

All the sensory disturbances of the control group were completely resolved within 10 weeks, and partial motor deficit was completely resolved by the fifth month. But for complete nerve injury, we performed an urgent revision surgery with STO and decreased sciatic nerve tension which resulted in recovery in nerve function immediately in 1 patient. Only 1 of the complete sciatic nerve lesions showed no recovery.

The difference of rate of sciatic nerve injury was not statistically significant between the 2 study groups (*P* = .08).

In the monitoring group, 11 (6.0%) patients had nerve dysfunction alert during surgery and couldn’t move their toes actively. All of them occurred in the process of reduction after the prosthetic trials were implanted, with the solutions including reducing the length of femoral neck (2 cases) trials and STO (9 cases) (Table 1).

**Table 1:** Summary of intraoperative and postoperative outcomes in patients undergoing total hip arthroplasty (THA) with intraoperative nerve monitoring (IONM) compared to a control group.

Parameter	Monitoring group (n = 183)	Control group (n = 156)
Postoperative nerve injury	None	6 patients (3 only peroneal, 3 both peroneal and tibial)
Complete nerve injury	0 cases	2 cases
Partial motor deficit	0 cases	3 cases
Partial motor & sensory deficit	0 cases	1 case
Nerve dysfunction alerts (during surgery)	11 patients (9 cases required subtrochanteric osteotomy)	Not applicable
Mean leg lengthening (range)	4.2 cm (2.4-5.6)	3.56 cm (2.2-5.6) (*P* value = .04)
Subtrochanteric trochanteric osteotomy	25 cases, 9 caused by nerve dysfunction	43 cases (*P* value < .005)

### Other outcomes

The mean achieved degree of leg lengthening was significantly greater in the monitoring group as 4.2 cm (range = 2.4-5.6) than in the control group as 3.56 cm (range = 2.2-5.6) (*P* = .04). The rate of STO was significantly lower in the monitoring group (13.6%, 25/183) compared to the control group (27.5%, 43/156) (*P* < .005). In the monitoring group, 25 patients underwent STO, and 9 of these osteotomies were caused by intraoperative nerve dysfunction. No other complications occurred in the 2 groups during the follow-up period.

## Discussion

We investigated the results of the active IONM by operating severe DDH patients under epidural anesthesia and found promising results for this approach compared to the cohort of patients without a nerve monitoring. The main findings of this study were the lower rates of nerve injury during THA, the greater extent of mean limb lengthening during surgery, and the lower rates of STO in patients with nerve monitoring while being under epidural anesthesia and actively been asked to move their toe during surgery.

Since the exactly similar studies to the current investigation are rare in literature, we tried to compare our findings with the most similar publications in the field to assess the achieved results. Chen et al. reported the results of their investigation on the use of wake-up test in 22 THA surgeries in 20 patients with high-riding DDH Crowe type Ⅳ, in which inability of foot dorsiflexion in wake-up test happened in only 1 patient reflecting the nerve injury that led to immediate reduction of the limb lengthening by 1 cm, and patient had drop foot and numbness for 2 months after surgery and all symptoms were resolved thereafter [13]. Also, all the other patients in their study were eventless after surgery and no neural deficits indicating nerve injury were detected, which led to the authors’ suggestion of this test as a simple and safe alternative to neurophysiologic IONM during THA in patients with severe DDH [13].

In a study be Kong et al. on patients with DDH Crowe type Ⅳ who underwent THA in 2 groups of 35 patients with IONM of the femoral and sciatic nerves and 56 patients in a historical controlled group without nerve monitoring, during the surgery of the monitoring group, 10 alerts happened and no neural complications were recorded after surgery, while in the control group 6 (10.7%) patients had neural complications postoperatively [12]. Also, the extent of limb lengthening was significantly greater in patients with nerve monitoring similar to our study (4.14 vs 3.64 cm, *P* = .04). Besides, the rate of STO was much lower in the monitoring group (17.1%) compared to the control group (58.9%) (*P* < .005), which were in consistency with our findings [12].

Shemesh et al. investigated the results of IONM to assess the sciatic nerve injuries in a series of 11 hip surgeries in 9 patients with severe DDH (Crowe types Ⅲ and Ⅳ) in which they stimulated the sciatic nerve before and after the relocation of the hip, with a mean of 28.5 mm leg lengthening (range = 6-51 mm), and reported 2 of 11 (18.2%) altered nerve response in stimulation after reduction that led to STO and femoral head size reduction [15]. The authors of that study suggested the use of handheld nerve stimulator as a tool for real-time assessment of nerve injury that helps the decision-making during surgery and preventing major nerve injuries in complex hip injuries [15].

In a study by Vanlommel et al. on 3 patients with 4 hips affected by Crowe types Ⅲ DDH who underwent THA via direct anterior approach and interoperative neurophysiological nerve monitoring was conducted to check the motor and somatosensory functions of the sciatic nerve, the mean leg lengthening was 24 mm (range = 20-36), and none of the cases had signs or symptoms of acute or chronic nerve damage in a mean follow-up period of 24 months [16]. The investigators of that study concluded that applying IONM enables surgeons to achieve significant limb lengths without neural injuries and postoperative complications [16].

While numerous studies highlight the efficacy of IONM in bony structures during hip and pelvic surgeries, a comprehensive systematic review of 32 studies underscores the ongoing controversy and debate surrounding its application. The review emphasizes the diverse array of IONM methods, each with specific advantages and disadvantages, coupled with varying sensitivity and specificity. This heterogeneity complicates the establishment of a universal guideline for IONM use across different surgical cases [18]. However, the general agreement is on the expansion of IONM methods to prevent sciatic nerve injuries during harsh maneuvers and positioning of limb in complex surgeries of hip like THA especially in complicated cases like severe DDH [18]. The less invasive approach, as demonstrated in this study through active toe movement, holds promise for detecting nerve injuries during surgery. Subsequent research endeavors may further solidify the utility of these methods in clinical practice.

The present study used the epidural anesthesia as a novel to keep the patient awake and enable assessment of the sciatic nerve function during surgery. In a large-scale long-term investigation of the rates of perioperative nerve injury in patients undergoing THA surgery based on the type of applied anesthesia, the overall rate of nerve injury was 0.72% (93 of 12,998) during 20 years and this rate was not associated with type of anesthesia [19]. As the authors of the mentioned study found that regional anesthesia methods like neuraxial anesthesia and peripheral nerve blockade were as safe as general anesthesia and the neurological recovery was independent of type of anesthesia, they recommended the use of regional anesthesia methods for candidates of elective THA considering the clinical benefits of these methods over more general methods [19].

The present study had some limitations. First, the study design was retrospective that limited the follow-up, specifically tracking neuromuscular force grading during the recovery from neural injuries. Second, there is an obvious trend toward reduced nerve injury with the aid of IONM which might have affected the results of the monitoring cohort in this study. Third, the lack of randomization and blinding might lead to selection bias. Additionally, the surgeon operating on patients possibly gained more awareness of nerve injury with increasing experience and took some extra precautions regardless of monitoring which could be associated with lower nerve injuries. In contrast, this study had several strengths like including a significant sample size that makes our findings more valid compared to similar studies, and using a novel technique for IONM by application of epidural anesthesia. Further studies using these anesthesia and surgery methods are suggested to validate the findings of this study and provide the evidence for future possible trials to prove its efficacy and safety.

## Conclusions

Active IONM via toe movement by patient under epidural anesthesia for THA with a severe DDH diagnosis is a viable option to timely detect and capture the sciatic nerve injury as this method could noticeably reduce the rates of neural damages and need for STO, and increase the degree of leg lengthening, that suggests this method as an alternative method to be used during complex hip surgeries.

## Funding statement

No specific grants were awarded for this study by any government, corporate, or nonprofit organizations.

## Conflicts of interest

The authors declare there are no conflicts of interest.

For full disclosure statements refer to https://doi.org/10.1016/j.artd.2024.101612.

## Ethics approval

This study received ethical approval from the Shahid Beheshti University of Medical Sciences (code: IR.SBMU.RETECH.REC.1402.356). Written informed consent was obtained from all participating patients prior to their inclusion in the study.

## CRediT authorship contribution statement

**Alireza Manafi Rasi:** Supervision, Conceptualization. **Sina Afzal:** Writing – review & editing, Writing – original draft, Software, Formal analysis. **Mojtaba Baroutkoub:** Investigation. **Hasan Shakiba:** Investigation. **Pooya Kalani:** Conceptualization. **Mehdi Tavassoli:** Investigation. **Reza Zarei:** Supervision.

## Availability of data and material

The data that support the findings of this study are available from Shahid Beheshti University of Medical Sciences, but restrictions apply to the availability of these data, which were used under license for the present study and so are not publicly available. Data are, however, available from the corresponding author upon reasonable request and with permission of Shahid Beheshti University of Medical Sciences.

## Appendix A Supplementary data

Conflict of Interest Statement for Rasi

Conflict of Interest Statement for Shakiba

Conflict of Interest Statement for Baroutkoub

Conflict of Interest Statement for Tavassoli

Conflict of Interest Statement for Kalani

Conflict of Interest Statement for Zarei

Conflict of Interest Statement for Afzal
